# 1Unilateral Le Fort I Advancement Versus Dentoalveolar Transport Distraction in Patients With Large Alveolar Clefts: Protocol for a Prospective Observational Study

**DOI:** 10.2196/77210

**Published:** 2026-08-25

**Authors:** Kundan Shibjee Jha, Nitin Bhola

**Affiliations:** 1 Department of Oral and Maxillofacial Surgery, Sharad Pawar Dental College, Datta Meghe Institute of Higher Education and Research, 04, SPDCH Bldg, Wardha, Maharashtra, 442107, India, 91 6393523675

**Keywords:** alveolar cleft, cleft lip and palate, tooth-borne distraction, segmental osteotomy advancement, graftless cleft repair

## Abstract

**Background:**

Cleft lip and palate is a complex congenital defect that can present functional and esthetic challenges, particularly in patients with large alveolar clefts for which surgical management is indicated. In contrast to traditional treatment techniques such as autologous bone grafting, newer techniques include unilateral Le Fort I osteotomy advancement and dentoalveolar transport distraction osteogenesis and have been shown to be useful in the treatment of maxillary hypoplasia and large alveolar clefts. These techniques may help improve both esthetic and function in patients with substantial tissue deficiencies as well as in patients with scarring.

**Objective:**

This study aims to compare the outcomes of unilateral Le Fort I advancement and dentoalveolar transport distraction in the management of large alveolar clefts, specifically focusing on the closure of alveolar gaps, maxillary arch form, stability of the advanced or distracted segment, and wound healing.

**Methods:**

This prospective observational study is being conducted at the Department of Oral and Maxillofacial Surgery, Siddharth Gupta Memorial Cancer Hospital, in collaboration with the Sharad Pawar Dental College in Maharashtra, India. Patients aged 9 to 25 years with unilateral alveolar clefts measuring 1 cm or greater will be included. Patients with smaller clefts, bilateral clefts, or those who are unsuitable for surgery will be excluded. The study will evaluate 14 patients, divided into 2 groups: group A will undergo dentoalveolar transport distraction, whereas group B will undergo unilateral Le Fort I advancement. We will assess the closure of the alveolar gap, arch form, stability of the advanced or distracted segment, and soft tissue healing. Through cone beam computed tomography and maxillary occlusal view, radiographic parameters of closure of the alveolar gap and arch form will be evaluated. The outcome measures will be statistically analyzed on RStudio software, with appropriate tests applied to each outcome measure. *P*<.05 will be considered significant.

**Results:**

As of June 2025, institutional ethical clearance has been obtained and patient recruitment is ongoing. Recruitment began in April 2023, with 12 patients enrolled as of June 2025. Data collection is expected to be completed by December 2025, and results will be published in mid-2026.

**Conclusions:**

This study aims to provide evidence-based guidance for the management of large alveolar clefts by comparing 2 graftless surgical techniques: unilateral Le Fort I advancement and dentoalveolar transport distraction. The findings are expected to help clinicians select the most suitable surgical approach based on individual case requirements, cleft morphology, and long-term outcome expectations. Ultimately, the study will contribute to optimizing cleft care strategies with reduced donor site morbidity and improved postoperative stability.

## Introduction

Cleft lip and cleft palate are among the most common congenital craniofacial anomalies, occurring in approximately 1 in every 700 live births globally, with variability based on ethnicity, gender, and geography [1]. These anomalies arise from the failure of normal fusion of facial processes during early intrauterine life and can occur in isolation or combination, unilaterally or bilaterally [2]. Alveolar clefts, often accompanying cleft lip or palate, result from a failure in primary palate formation, leading to discontinuity in the maxillary arch. This disrupts dental development, speech, mastication, and nasal function and predisposes individuals to oronasal fistulae [3].

The traditional method for alveolar cleft management has been secondary alveolar bone grafting (SABG), introduced by Boyne and Sands in 1976 [4]. SABG aims to bridge the cleft with autogenous cancellous bone, typically from the iliac crest, to allow tooth eruption and stabilize the arch. Despite its widespread use, SABG presents limitations, especially in large clefts, due to issues such as donor site morbidity, unpredictable graft resorption, and soft tissue insufficiency leading to wound dehiscence [5,6]. These drawbacks have led to the exploration of graftless alternatives such as distraction osteogenesis (DO).

DO, first applied to the craniofacial skeleton by McCarthy et al (1992) [7], involves gradual movement of bone segments to promote new bone formation. Dentoalveolar transport distraction, a subset of DO, has demonstrated effectiveness in reducing cleft width and promoting both bone and soft tissue regeneration, particularly in wide alveolar defects where bone grafting may fail [8-10]. Unilateral Le Fort I advancement, another surgical technique, enables forward repositioning of the cleft-side maxilla to close the defect while simultaneously correcting midface retrusion [11]. Both techniques offer graftless solutions with promising outcomes, although comparative clinical data between them remain limited.

Considering the complexity of large alveolar clefts and the limitations of conventional bone grafting, this study aims to evaluate the outcomes of dentoalveolar transport distraction compared with unilateral Le Fort I advancement. The analysis will focus on cleft gap closure, maxillary arch form, the stability of mobilized section, and postoperative healing. This comparative assessment will offer sought-after information to direct surgical decision-making in the treatment of wide alveolar clefts, as the traditional techniques often fail due to graft resorption, soft tissue dehiscence, or inadequate bone regeneration.

While both unilateral Le Fort I advancement and dentoalveolar transport distraction have been independently used for managing large alveolar clefts, comparative clinical data between these 2 techniques are lacking. Current literature focuses on individual case series or retrospective studies, with no consensus on which method yields superior outcomes in terms of bone regeneration, stability, or soft tissue healing. Given the unpredictability and donor site morbidity of traditional grafting methods in wide clefts, a direct prospective comparison of these 2 graftless techniques is warranted. This study aims to fill that gap by systematically evaluating and comparing the clinical, esthetic, and radiographic outcomes of both procedures in patients with large alveolar clefts.

To guide the comparison between unilateral Le Fort I advancement and dentoalveolar transport distraction in the management of large alveolar clefts, the following hypotheses were formulated:

- Alternate hypothesis—there is a detectable difference between unilateral Le Fort I advancement and dentoalveolar transport distraction in at least 1 of the following outcomes: alveolar gap closure (measured radiographically), arch form (classified clinically and radiographically), stability of the mobilized segment (graded on the bone stability scale), or soft tissue healing (graded on the Landry wound healing scale) in patients with large alveolar clefts.
- Null hypothesis—there is no detectable difference between unilateral Le Fort I advancement and dentoalveolar transport distraction in alveolar gap closure, arch form, segment stability, or soft tissue healing outcomes in patients with large alveolar clefts.

## Methods

### Overview

This is a nonrandomized, prospective observational study conducted at the Department of Oral and Maxillofacial Surgery, Siddharth Gupta Memorial Cancer Hospital, in collaboration with Sharad Pawar Dental College, Maharashtra, India. The estimated study duration is from April 2023 to December 2025.

A total of 17 patients have been screened, of whom 70.58% (12/17) participants have met the eligibility criteria and have been enrolled. Patients have been allocated to 1 of 2 intervention groups (dentoalveolar transport distraction or unilateral Le Fort I advancement) based on individualized clinical considerations, including cleft morphology, available bone stock, and expected compliance with the treatment protocol. Allocation was determined by the treating maxillofacial surgeon in accordance with predefined feasibility criteria.

### Ethical Considerations

This study was reviewed and approved by the Institutional Ethics Committee of Datta Meghe Institute of Higher Education and Research (DMIHER(DU)/IEC/2023/843). All procedures performed will be in accordance with the ethical standards of the responsible institutional committee and with the principles of the Declaration of Helsinki. Written informed consent has been obtained from all participants (or their legal guardians when applicable) prior to inclusion in the study. Participant data will be anonymized, and confidentiality and privacy of all individuals will be maintained according to institutional and national regulations. No personal identifying information will be disclosed. No compensation will be provided to participants for their involvement in the study.

### Intervention Delivery

All surgeries will be performed by experienced oral and maxillofacial surgeons under general anesthesia.

The surgical procedures for both study groups will be standardized and carried out according to established protocols, as outlined below:

- In *group A (dentoalveolar transport distraction)*, after adequate orthodontic preparation and osteotomy, a custom-fabricated intraoral distractor was placed. Distraction commenced after a latency period of 5 to 7 days, at a rate of 1 mm per day until gap closure, followed by a consolidation period of 8 to 12 weeks.
- In *group B (unilateral Le Fort I advancement)*, standard Le Fort I and palatal osteotomies were performed. The cleft-side maxillary segment was repositioned medially and stabilized using a surgical splint and titanium plates.

Postoperative care will be standardized across both groups and included antibiotics, analgesics, antiseptic rinses, and dietary modifications.

### Outcome Measures and Data Collection

Data collection will occur at predefined intervals: preoperatively; immediately after surgery; and at 1, 3, 6, and 12 months of follow-up.

The following outcome variables will be systematically evaluated in both groups:

- Alveolar gap closure will be measured radiographically using cone beam computed tomography (CBCT) at each interval.
- Maxillary arch form will be classified (U-shaped, V-shaped, or asymmetrical) using dental models and occlusal views.
- Segment stability will be assessed clinically and via CBCT using the 5-point bone stability scale.
- Soft tissue healing will be evaluated by blinded assessors using the Landry wound healing scale.

All CBCT images will be anonymized and randomized. Two independent evaluators, blinded to group assignment, will perform measurements. If visible hardware reveals the treatment allocation, a second reviewer re-evaluated the image independently.

### Feasibility and Acceptability

Feasibility will be monitored by tracking the completion of surgical procedures, follow-up adherence, and compliance with distraction protocols. Acceptability will be inferred based on the absence of withdrawals and participants’ tolerance to the intervention schedule. As of June 2025, no participant dropouts or major protocol deviations have been recorded.

### Primary Objective

The primary objective will be to compare the outcomes of unilateral Le Fort I advancement and dentoalveolar transport distraction in the closure of alveolar gap, arch form, segment stability, and soft tissue healing.

### Secondary Objectives

The secondary objectives will be to assess the intragroup outcomes over time for each technique and to evaluate patient-specific variables (eg, age and cleft width) that may influence outcome variation.

### Sample Size Calculation

The minimum required sample size was calculated using the standard formula for comparing 2 independent means, as shown below:


$$N=\frac{{\left(Z_{\alpha }+Z_{\beta }\right)}^{2}\left(P_{1}\left(1-P_{1}\right)+P_{2}\left(1-P_{2}\right)\;\right)}{{\left(P2-P1\right)}^{2}}$$

$$Z_{\alpha }=1.64$$

α= Type I error at 5%

$$Z_{\beta }=0.84$$

β= Type II error at 20%

The following parameters were used for the sample size calculation:

- Improved facial esthetic and balance in maxillary DO=94%
- Improved facial esthetic and balance in maxillary unilateral Le Fort 1 osteotomy=44% (estimated clinically significant difference=50%)
- Difference (P2−P1)=50%
- Minimum sample size N=2×(1.64+0.84)^2^ (0.94) (1‐0.94) + 0.44(1‐0.44)/(0.50)^2^=7 each in 2 groups
- Sample size=14, with 7 in each group

Using a 2-sided z test with *α*=.05 and power of 80%, assuming a success rate of 94% in the dentoalveolar transport distraction group and 44% in the unilateral Le Fort I advancement group, the required sample size is 7 per group (N=14). This includes a 50% absolute effect size and accounts for variability in response.

The 50% effect size estimate was based on the internal pilot data and clinical observations. However, we acknowledge the possibility of overestimation. A sensitivity analysis will also consider a 40% effect size, which would require a larger sample and will be addressed in future studies.

### Primary Outcome

The primary outcomes evaluated in this study will be as follows:

- Closure of the alveolar gap: measured clinically and radiographically (ie, CBCT) at 5 time points: preoperatively; immediately after surgery; and at 1, 3, and 6 months and 1 year of follow-up
- Arch form: classified (U-shaped, V-shaped, or asymmetrical) via models and radiographs at each follow-up

### Secondary Outcomes

The secondary outcomes assessed in this study will include the following:

- Stability of the advanced or transported segment: evaluated by bidigital palpation and CBCT and graded on a 5-point bone stability scale (1=highly unstable; 5=highly stable).
- Soft tissue healing: evaluated by the Landry wound healing scale at all times of follow-up and graded from 1 (very poor) to 5 (excellent).

All outcome assessments will be performed by evaluators who are blinded to the treatment group. CBCT scans will be anonymized, coded, and randomized before analysis. In cases where unblinding may occur (eg, visible hardware on radiographs), a second independent blinded reviewer will reassess.

### Preoperative Assessment

All patients will undergo clinical examination and medical history review. Informed consent will be obtained. The interdental cleft width will be measured using Castroviejo calipers, and arch form will be classified. CBCT, lateral cephalograms, and maxillary occlusal radiographs will be performed to assess the cleft.

### The Dentoalveolar Transport Distraction Group

Orthodontic spacing will be created between the premolars. A horizontal mucosal incision will be made, followed by horizontal, anterior, posterior, and interdental osteotomies. A custom-made intraoral distractor will be fixed. After a 5- to 7-day latency period, distraction will begin at 1 mm per day until cleft closure. A consolidation period of 8 to 12 weeks will follow.

### The Unilateral Le Fort I Advancement Group

Orthodontic preparation will be done similarly. A horizontal incision will be made, and a mucoperiosteal flap will be raised to expose the anatomical landmarks. Standard Le Fort I and palatal osteotomies will be carried out. The segment will be medially repositioned and fixed using a surgical splint and bone plates. The flap will be closed with 3‐0 Vicryl sutures.

### Postoperative Care

All patients will receive antibiotics, analgesics, chlorhexidine mouth rinses, and dietary instructions. Follow-up visits will be scheduled at 1 month, 3 months, 6 months, and 1 year postoperatively.

As this is a prospective observational study, treatment allocation is not randomized. Patients are assigned to either the dentoalveolar transport distraction group or the unilateral Le Fort I advancement group based on specific clinical indications determined by the treating maxillofacial surgeon. Criteria such as cleft morphology, cleft width, presence of adequate bone stock for distraction, soft tissue quality, and anticipated compliance with the distraction protocols are considered during group assignment. To minimize selection bias, all assessments are conducted using standardized protocols, and outcome evaluators are blinded to the intervention type. Additionally, baseline characteristics of both groups will be statistically compared to assess equivalency, and any confounders will be adjusted for during analysis using multivariate regression techniques.

To further minimize allocation bias inherent to the nonrandomized design, we will perform stratified enrollment based on cleft width and age groups. Additionally, a propensity score adjustment will be conducted in the final analysis to balance any observed confounders between groups.

Missing data may arise from loss to follow-up or incomplete clinical or radiographic records. These will be assessed for randomness using the Little missing completely at random test. If data are missing at random, multiple imputation using chained equations will be applied to minimize bias. Variables with >20% missing data will be excluded from the primary analysis but may be used in sensitivity analysis. Potential confounders, such as patient age, cleft width at baseline, and compliance with treatment protocols, will be identified a priori based on clinical relevance and literature review. These variables will be included in multivariate regression models (eg, linear regression for continuous outcomes such as gap width and logistic regression for binary outcomes such as successful closure) to adjust the primary effect estimates. Adjusted effect sizes and CIs will be reported alongside unadjusted comparisons.

### Statistical Analysis

All data will be analyzed using RStudio (version 4.3.2; Posit, PBC). Continuous variables (eg, cleft width and bone segment stability score) will be tested for normality using the Shapiro-Wilk test. Depending on data distribution, between-group comparisons will be performed using either the unpaired *t* test (for normally distributed data) or the Mann-Whitney *U* test (for nonparametric data). Categorical outcomes, such as arch form classification (U-shaped, V-shaped, or asymmetrical) and wound healing grades (from the Landry wound healing scale), will be compared using the chi-square test or Fisher exact test, as appropriate.

Longitudinal outcomes (eg, segment stability across time points) will be analyzed using repeated measures ANOVA for normally distributed data or generalized estimating equations for nonparametric repeated measures. All outcome measures will be reported with 95% CIs, and *P*<.05 will be considered statistically significant. Missing data will be handled using multiple imputation, and any potential confounders, such as age or initial cleft width, will be adjusted for using multivariate regression analysis.

To assess the robustness of results, sensitivity analysis will be conducted by comparing results from multiple imputation with complete-case analysis and worst-case scenario imputation.

## Results

As of June 2025, patient recruitment, which began in April 2023, is ongoing. Out of 14 intended participants, 12 (85%) have been enrolled (with 7 in the dentoalveolar transport distraction group and 5 in the unilateral Le Fort I advancement group). The enrolled cohort includes 8 male individuals and 4 female individuals, with a mean age of 16.8 (SD 3.9; range 10-23) years. The average cleft width among the participants was 1.5 (SD 0.34; range 1.0-2.1) cm.

Data collection is anticipated to conclude by December 2025, and the results are expected to be published in 2026.

## Discussion

### Anticipated Findings

This prospective observational study compares 2 graftless surgical techniques—unilateral Le Fort I advancement and dentoalveolar transport distraction—for the management of large alveolar clefts. On the basis of prior evidence, we anticipate that dentoalveolar transport distraction may achieve more predictable closure of wide alveolar gaps due to its capability for simultaneous bone and soft tissue regeneration through gradual mechanical tension [8,9]. Unilateral Le Fort I advancement, while traditionally used for midfacial hypoplasia and skeletal advancement, may offer improved esthetic facial projection but may be limited by the extent of cleft closure, particularly in large defects [10]. This study will evaluate and compare the clinical, radiographic, and soft tissue healing outcomes of both interventions to identify their respective advantages.

Several studies have demonstrated the effectiveness of dentoalveolar transport distraction in achieving successful cleft closure, even in patients for whom conventional bone grafting had failed [8,9,12]. These techniques exploit the principle of DO, first introduced to the craniofacial skeleton by McCarthy et al [7] and later adapted to the alveolar ridge [8]. Similarly, Le Fort I osteotomy, first described for cleft-related maxillary retrusion, has shown good results in terms of facial profile improvement and occlusion correction [11]. However, few prospective studies have evaluated these 2 methods head-to-head in a controlled population with similar cleft widths, making direct clinical comparison difficult. This study aims to address this gap by applying standardized clinical and radiographic outcome measures across both groups.

The study’s strengths include its prospective design, structured follow-up, and the use of objective evaluation tools, including CBCT for gap measurements and standardized scales such as the Landry wound healing scale and the bone stability scale. By focusing on large clefts (≥1 cm) and excluding patients with bilateral defects or prior interventions, the study enhances internal validity. Limitations include the relatively small sample size, which may limit generalizability, and the nonrandom allocation of patients to treatment groups based on clinical feasibility. However, multivariate regression will be used to adjust for baseline differences, and standardized surgical and postoperative protocols will minimize procedural variability.

Although not formally implemented in this study, future work could explore integrating adaptive patient-reported outcome measures such as the Cleft Lip and Palate Patient-Reported Outcome Measure platform to assess quality of life and surgical satisfaction [13].

Depending on the outcomes, this study may inform clinical treatment pathways for managing wide alveolar clefts using graftless techniques. Future research could expand to include long-term assessment of relapse, dental eruption across the regenerated cleft, and patient-reported outcome measures such as quality of life and esthetic satisfaction. Integration with 3D simulation and planning tools could further improve precision in surgical execution.

Recent reviews and bibliometric analyses show a marked increase in research on maxillary distraction protocols, reflecting growing global interest in optimizing cleft management [14].

Findings from this study will be submitted for publication in peer-reviewed journals and presented at national and international maxillofacial surgery conferences. Additionally, results will be shared with our institution’s multidisciplinary cleft care teams to inform clinical decision-making. If substantial differences are observed, the study may contribute to the development of evidence-based treatment guidelines for wide alveolar clefts.

### Conclusions

This study protocol describes a prospective, structured comparison of the unilateral Le Fort I advancement and dentoalveolar transport distraction in patients with large alveolar clefts. By evaluating key outcomes such as cleft gap closure, arch form, soft tissue healing, and segment stability, the study aims to provide evidence-based guidance for surgical management where conventional grafting techniques may be less effective [5,6]. While results are pending, this protocol sets the groundwork for standardized outcome evaluation in cleft surgery and introduces a comparative framework for 2 promising graftless techniques.

## Abbreviations

CBCT: cone beam computed tomography
DO: distraction osteogenesis
SABG: secondary alveolar bone grafting
